# Supplementary material for: Depolymerisation of Kraft Lignin by Tailor-Made Alkaliphilic Fungal Laccases
Source: Polymers (Basel). 2023 Nov 16;15(22):4433. doi: 10.3390/polym15224433 (PMC10675753; doi:10.3390/polym15224433)
Supplement: Supplementary file 1 [file polymers-15-04433-s001.zip › polymers-2682592-supplementary.pdf]

Supplementary data

Depolymerisation of kraft lignin by tailor-made alkaliphilic fungal laccases

David Rodríguez-Escribano, Felipe de Salas, Rocío Pliego, Gisela Marques, Thomas Levée, Anu Suonpää, Ana Gutiérrez, Ángel T. Martínez, Petri Ihalainen, Jorge Rencoret* and Susana Camarero*.

Table S1. Mass balance of lignin fractions generated after 24 h-reaction of eucalyptus kraft lignin (25 g/L) with the alkaliphilic fungal laccase (C-LeB) or without (control) at pH 10, followed by sample drying and resuspension in HCl. Percentages are relative to the initial lignin amount.

Lignin	Control (mg)	Laccase (mg)
Aqueous phase (A) pH 10		
Acid insoluble fraction	480 (20%)	735 (28%)
Acid soluble fraction	619 (27%)	218 (8%)
Total	1099 (47%)	953 (36%)
Solid phase (S) pH 10		
Acid insoluble fraction	1100 (47%)	1690 (63.5%)
Acid soluble fraction	139 (6%)	13 (0.5%)
Total	1239 (53%)	1703 (64%)
Total (A+S)	2338 (100%)	2656 (100%)

*The total lignin (mg) is calculated by the sum of weights of the precipitated lignins and the amounts of soluble lignins estimated from the Abs at 205 nm.

Table S2. Average Mw (Da) and polydispersity (PDI) values determined by GPC, and content (mmol/g) in phenolic (PhOH), carboxylic (COOH) and aliphatic (AliphOH) hydroxyl groups determined by ^{31}P-NMR, of permeate and retentate fractions obtained from the treatment of Eucalyptus kraft lignin with the alkaliphilic fungal laccase (5 U Li10 /g of lignin) in the bioreactor coupled to a membrane separation system.

	2.6-DMP	Vanillin	Acetovanillone	Syringaldehyde	Vanillylpropanol	Benzoic Acid	Syringic Acid	Total PhOH monomers (mmol/g total lignin)
C-Aas	8.5	1.8	2.5	1.7	1.0	1.4	5.2	14.9
L-Aas	0	0.6	0.6	4.4	0	0.3	0.3	1.2
C-Sas	1.2	0	0.4	0.5	0	0.2	0.4	0.5
L-Sas	0	0.2	0.2	1.3	0	0.7	0	Traces

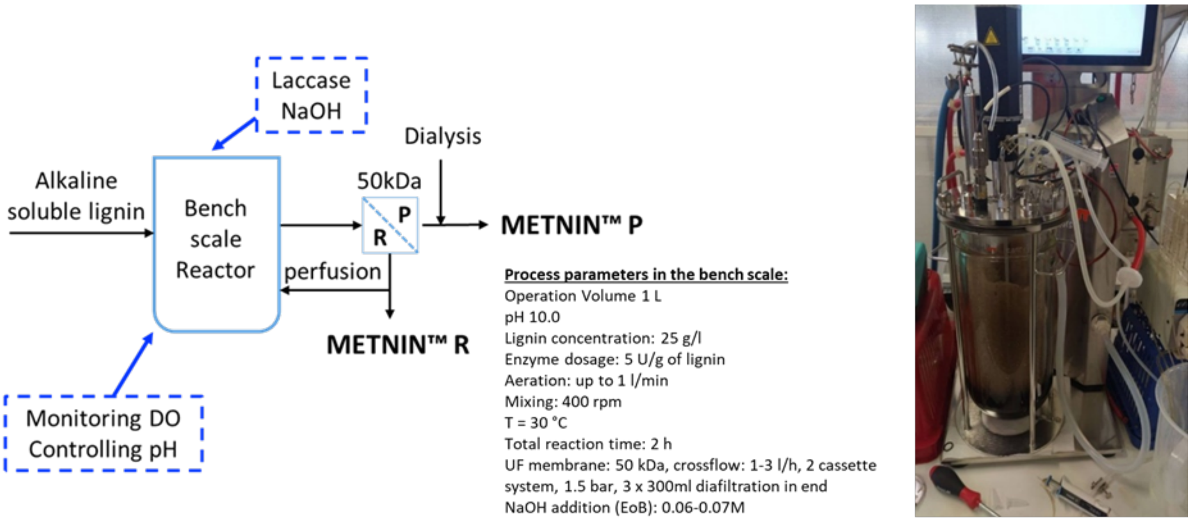

Figure S1. Scheme of the bench-scale set-up including the process parameters for the reaction with the alkaliphilic Li10 fungal laccase (left), and the bioreactor (7.5 l volume) coupled to a membrane separation system (right).

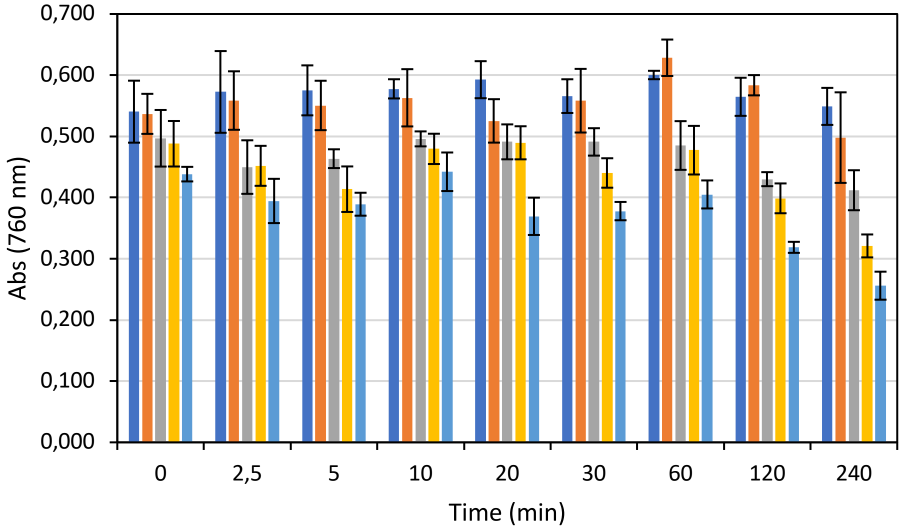

Figure S2. Changes in the phenolic OH content (measured by FCR) of Eucalyptus kraft lignin after treatment with different concentrations of C-LeB laccase for 2.5- 240 min. From left to right bars represent: control without enzyme (0), 6, 30, 60 and 180 mU/mg.
